# Epidemiological and clinical characteristics of patients with suspected COVID-19 admitted in Metro Manila, Philippines

**DOI:** 10.1186/s41182-020-00241-8

**Published:** 2020-06-22

**Authors:** Eumelia P. Salva, Jose Benito Villarama, Edmundo B. Lopez, Ana Ria Sayo, Annavi Marie G. Villanueva, Tansy Edwards, Su Myat Han, Shuichi Suzuki, Xerxes Seposo, Koya Ariyoshi, Chris Smith

**Affiliations:** 1 San Lazaro Hospital, Manila, Philippines; 2 School of Tropical Medicine and Global Health, Nagasaki University, Nagasaki, Japan; 3 MRC Tropical Epidemiology Group, London School of Hygiene and Tropical Medicine, London, UK; 4 Institute of Tropical Medicine, Nagasaki University, Nagasaki, 852-8523 Japan; 5 Faculty of Infectious and Tropical Diseases, London School of Hygiene and Tropical Medicine, London, UK

**Keywords:** COVID-19, SARS-CoV-2, Coronavirus, Philippines, Manila, Epidemiology, Case fatality rate

## Abstract

**Background:**

Coronavirus disease 2019 (COVID-19) has spread to almost every region and country in the world, leading to widespread travel restrictions and national lockdowns. Currently, there are limited epidemiological and clinical data on COVID-19 patients from low and middle-income countries. We conducted a retrospective single-center study of the first 100 individuals with suspected COVID-19 (between Jan. 25 and Mar. 29, 2020) admitted to San Lazaro Hospital (SLH), the national infectious diseases referral hospital in Manila, Philippines.

**Results:**

Demographic data, travel history, clinical features, and outcomes were summarized and compared between COVID-19 confirmed and non-confirmed cases. The first two confirmed cases were Chinese nationals, admitted on Jan. 25. The third confirmed case was a Filipino, admitted on Mar. 8. Trends toward confirmed COVID-19 cases not reporting international travel and being admitted to SLH from the densely populated area of Manila city were observed during Mar. 8-29. All 42 of the 100 confirmed COVID-19 cases were adults, 40% were aged 60 years and above and 55% were male. Three were health workers. Among individuals with suspected COVID-19, confirmed cases were more likely to be older, Filipino, not report international travel history and have at least one underlying disease, particularly diabetes, report difficulty in breathing, and a longer duration of symptoms. In over 90% of non-COVID-19 cases, the alternative diagnosis was respiratory. Nine (21%) confirmed cases died. The median duration from symptoms onset to death was 11.5 (range: 8–18) days.

**Conclusions:**

Imported COVID-19 cases have reduced but local transmission persists and there is a trend toward cases being admitted to SLH from densely populated areas. This study highlights the difficulty in diagnosing COVID-19 on clinical grounds and the importance of diagnostic capacity in all settings. Difficulty of breathing was the only symptom associated with COVID-19 infection and should alert clinicians to the possibility of COVID-19. Clinical characteristics of confirmed COVID-19 cases and a hospital case fatality rate of 21% are comparable with other settings.

## Introduction

Coronavirus disease 2019 (COVID-19) is a respiratory disease caused by a novel coronavirus named severe acute respiratory syndrome coronavirus 2 (SARS-CoV-2) [1]. Since December 2019, when initial cases were identified in Wuhan, China, COVID-19 has spread to almost every region and country in the world, leading to widespread travel restrictions and national lockdowns [1]. The World Health Organization declared a global pandemic on Mar. 11, 2020 [2]. The epidemiological and clinical characteristics of COVID-19 have been well described in a range of settings, including China, the USA, and Singapore [3–5]. However, there are limited reports of the epidemiological and clinical characteristics of COVID-19 from low and middle income (LMIC) countries with tropical climates.

The first COVID-19 case in the Philippines was confirmed on Jan. 31, 2020, admitted to San Lazaro Hospital (SLH), the national infectious disease referral hospital in Manila [6]. The second case was the close contact of the first case and was the first confirmed death in the country as well as the first mortality outside China. Both of the cases were imported cases (travelers from China). The Philippine government implemented travel restrictions on foreign travelers from Hubei province on Jan. 31, and then extended this to include additional countries with COVID-19 cases during February [7–9]. The first confirmed local transmission of COVID-19 in the country, without any travel history, was reported on Mar. 5 [10]. As of May 2, 8772 confirmed cases were reported in the Philippines [11]. In this study, we describe the epidemiological, clinical characteristics, and clinical outcomes of the first 100 individuals with suspected COVID-19 admitted to SLH by Mar. 31, 2020.

## Methods

### Study design and participants

We conducted a retrospective single-center descriptive study summarizing the first 100 individuals with suspected COVID-19 admitted to SLH, which serves a low-income population in Manila, the most densely populated city with Metropolitan Manila, Philippines. During this time, adult and pediatric patients with suspected COVID-19 residing in the National Capital Region of Manila were admitted to SLH, in addition to other hospitals dedicated for COVID-19 admission in the region. Admitted patients included either self-referrals (walk-in patients) or referrals from other health facilities through direct coordination or through the Regional Epidemiologic Surveillance Unit (RESU). From March 7, the Department of Health (DOH) guidelines stated that only confirmed COVID-19 patients that were severe or critical should be referred to SLH [12].

This was a retrospective analysis of anonymized routinely collected data. The study was approved by the SLH research ethics and review unit (Ref: SLH-RERU-2020-022-I) and the School of Tropical Medicine and Global Health, Nagasaki University Ethical Committee (NU_TMGH_2020_119_1).

### Procedures

We obtained data from “Case Investigation Forms (CIF)” for 2019 coronavirus disease completed by the clinical teams and submitted to the SLH epidemiology department. The CIF was designed by the Philippines DOH Epidemiology Bureau, which evolved from the SARI (severe acute respiratory illness) case report form, and collected information in the following domains: patient profile, Philippine residence, overseas employment address (if relevant), travel history, likely exposure (imported or local), clinical information, specimen information final classification (COVID-19 or not COVID-19), outcome (died or discharged). Information on whether the patient self-referred or was referred from another hospital was not systematically recorded. Selected data were encoded to create an anonymized dataset. Clarifications were discussed with the epidemiology department encoders or clinical teams. The analysis was undertaken after clinical outcomes were available for all patients.

### Case definition of COVID-19 suspect

The case definition for a suspected COVID-19 individual in the Philippines has been modified over a short period of time as the epidemic has evolved. The initial decision tool released by the DOH on Jan. 21, 2020 [13], classified individuals as a suspected case, or person under investigation (PUI), if they fulfilled at least three of the following criteria: fever, respiratory infection (cough and/or coryza), residence or travel history to Wuhan, Hubei, in the 14 days prior to symptom onset, or a history of exposure such as close contact with a confirmed case [14]. The case definition was adapted on Feb. 26, 2020, with the suspected COVID-19 case criteria expanded to include all areas with travel restrictions [15]. Following the onset of community transmission, on Mar. 16, the case definition was further modified to include individuals without a travel history and added shortness of breath to the symptom list [10]. Cases were considered “imported” if a history of international travel was reported within 14 days prior to the admission, and conversely “local” if no international travel was reported within 14 days prior to the admission.

### Confirmatory test for COVID-19

Laboratory confirmation for the first COVID-19 case was performed at the Victorian Medical Center (Australia) [16] and subsequently for all other cases at the Research Institute for Tropical Medicine (RITM) [17]. Nasopharyngeal and oropharyngeal swabs (NPS/OPS), and in some cases sputum and endotracheal aspirates, were obtained from patients and maintained in viral-transport medium. COVID-19 was confirmed by real-time PCR detecting SARS-CoV-2 at the RITM using the Corman et al. protocol [13].

### Statistical analysis

We summarized demographic characteristics, travel history, symptoms on admission, co-morbidities by whether a suspected case tested positive for COVID-19 or not. We also summarised duration between onset of symptoms and admission. Continuous variables were expressed as mean (standard deviation, SD), median (range), and categorical variables were expressed as number (%). Fisher’s exact test was used to test for associations between categorical variables and Mann-Whitney tests were used to compare discrete variables between categories of categorical variables. All analyses were performed using Stata v15 [18].

## Results

**Fig. 1:**
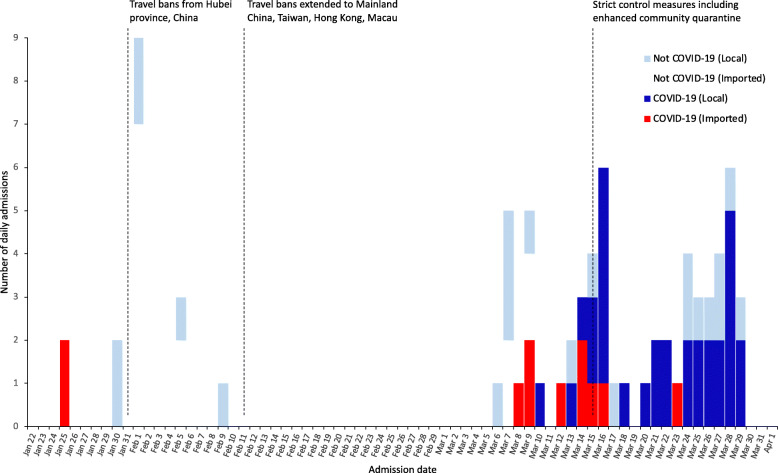
Timeline of admission date of the first 100 suspected COVID-19 cases to an infectious diseases hospital in Metro Manila. Cases were considered “imported” if a history of international travel was reported within 14 days prior to the admission, and conversely ‘local’ if no international travel was reported within 14 days prior to the admission

**Fig. 2:**
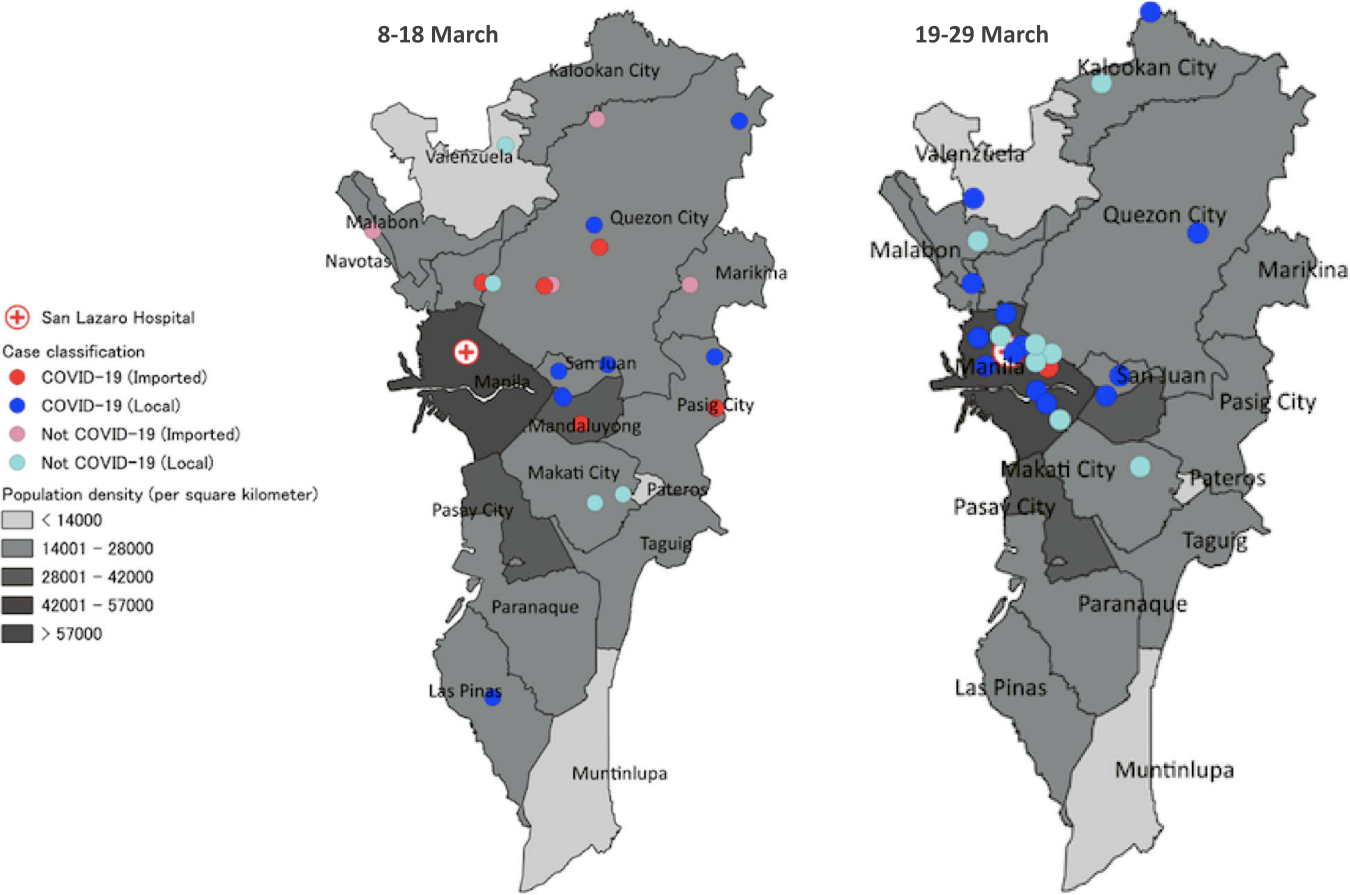
Residence of suspected and confirmed COVID-19 individuals if resident in the National Capital Region of Metropolitan Manila admitted during 8*–*18 March (left) and 19*–*29 March (right). Twenty-two individuals were admitted during Mar. 8*–*18, of whom 14 were confirmed COVID-19 (imported [5] vs. local [9]). Twenty-three individuals were admitted during Mar. 19*–*29 of whom 15 were confirmed COVID-19 (imported (*n* = 1) vs. local [*n* = 14]). Excludes population not resident in Metro Manila (*n* = 21) or admitted before March 8 (*n* = 34). Dots overlap in the case of identical or similar residence and hence a separate dot is not visible for every case

One hundred patients with suspected COVID-19 infection admitted in SLH from Jan. 25 to Mar. 29, 2020, were included in this study. Of these, 42 (42%) were identified as laboratory-confirmed COVID-19. Figure 1 shows the timeline of admission of cases indicating whether a history of international travel was reported. The first two suspected patients were admitted on Jan. 25, 2020, both of whom became confirmed cases. During Jan. 27-Mar. 7, a further 42 suspected cases were admitted, none of whom were confirmed cases. The next confirmed case was admitted on Mar. 8. From Mar. 8-29, a further 55 suspected cases were admitted, of whom 39 were confirmed cases. Figure 2 shows the approximate residence of suspected and confirmed COVID-19 cases admitted during Mar. 8-29, 2020, excluding those residents outside Metro Manila or staying in a hotel. During Mar. 8-18, 14 confirmed cases resident in Metro Manila were admitted to SLH, none of whom lived in Manila city. During Mar. 19-29, 15 confirmed cases resident in Metro Manila were admitted to SLH, of whom 8 lived in Manila city.

Table 1 shows the demographic and baseline characteristics of the 100 individuals with suspected COVID-19. Most were aged over 18 (98%) and Filipino (83%). Other nationalities included Chinese (14), American (2), and German (1). Just over half were male (58%), 11% were healthcare workers and recent travel history outside of the Philippines was reported in 47% of suspected cases. Around one-third of suspected cases reported at least one underlying disease (31%), including hypertension (23%), diabetes (9%), cardiovascular disease (7%), respiratory disease (10%), and HIV (1%). One patient reported being pregnant.

**Table 1:** Demographic and baseline characteristics of suspected COVID-19 patients

Characteristic		All	COVID-19	Not COVID-19	*p* value
Overall		100	42	58	
Age (years)	*N*	100	42	58	
	Mean (SD)	45 (19)	52 (16)	39 (19)	
	Median (range)	42.5 (4, 89)	56.5 (24, 89)	31.5 (4, 88)	< 0.001
Age group (years)	1–5	1 (1)	0 (0)	1 (2)	0.003
	6-12	0 (0)	0 (0)	0 (0)	
	13-18	1 (1)	0 (0)	1 (2)	
	19-40	47 (47)	12 (29)	35 (60)	
	41-59	25 (25)	13 (31)	12 (21)	
	60 +	26 (26)	17 (40)	9 (16)	
Sex	Female	42 (42)	19 (45)	23 (40)	0.682
	Male	58 (58)	23 (55)	35 (60)	
Nationality	American	2 (2)	0 (0)	2 (3)	0.024
	Chinese	14 (14)	2 (5)	12 (21)	
	Filipino	83 (83)	40 (95)	43 (74)	
	German	1 (1)	0 (0)	1 (2)	
Health worker	No	89 (89)	39 (93)	50 (86)	0.350
	Yes	11 (11)	3 (7)	8 (14)	
Recent international travel history (within 14 days prior to admission)	No	53 (53)	31 (74)	22 (38)	< 0.001
	China	19 (19)	3 (7)	16 (28)	
	other Asia	24 (24)	5 (12)	19 (33)	
	Europe or USA	3 (3)	2 (5)	1 (2)	
	United Arab Emirates	1 (1)	1 (2)	0 (0)	
Reported symptoms					
Coryza	No	59 (59)	24 (57)	35 (60)	0.838
	Yes	41 (41)	18 (43)	23 (40)	
Cough	No	30 (30)	10 (24)	20 (34)	0.277
	Yes	70 (70)	32 (76)	38 (66)	
Fever	No	61 (61)	24 (57)	37 (64)	0.538
	Yes	39 (39)	18 (43)	21 (36)	
Sore throat	No	79 (79)	30 (71)	49 (84)	0.139
	Yes	21 (21)	12 (29)	9 (16)	
Difficulty breathing	No	82 (82)	30 (71)	52 (90)	0.033
	Yes	18 (18)	12 (29)	6 (10)	
Other symptoms	No	94 (94)	39 (93)	55 (95)	0.694
	Yes	6 (6)	3 (7)	3 (5)	
Co-morbidities					
Cardiovascular disease	No	93 (93)	38 (90)	55 (95)	0.449
	Yes	7 (7)	4 (10)	3 (5)	
Diabetes	No	91 (91)	35 (83)	56 (97)	0.033
	Yes	9 (9)	7 (17)	2 (3)	
Hypertension	No	77 (77)	28 (67)	49 (84)	0.053
	Yes	23 (23)	14 (33)	9 (16)	
Respiratory illness, including asthma	No	90 (90)	35 (83)	55 (95)	0.090
	Yes	10 (10)	7 (17)	3 (5)	
HIV	Unknown	99 (99)	42 (100)	57 (98)	-
	Yes	1 (1)	0 (0)	1 (2)	
At least one underlying disease	No	69 (69)	22 (52)	47 (81)	0.004
	Yes	31 (31)	20 (48)	11 (19)	
Pregnancy in women		1/41 (3%)	0/19 (0%)	1/22 (4%)	-
Duration between onset of symptoms and admission	*N*	97	39	58	
	mean (SD)	6 (6)	8 (5)	5 (6)	
	median (range)	4 (0, 29)	7 (0, 19)	3 (0, 29)	< 0.001

Data are *n* (%) unless otherwise stated

*p* value from Fisher’s exact test for categorical variables or Mann-Whitney for continuous variables

Cough was the predominant symptom reported (70%), followed by coryza and fever in around 40%, then sore throat and difficulty of breathing in around 20% of individuals with suspected COVID-19. A small number (6%) had other symptoms which included back pain (2%), diarrhea (2%), and body malaise (2%). The median time from onset of symptoms to hospital admission was 4 days (0, 29).

All confirmed COVID-19 cases were adults. Confirmed cases were older (Fishers test *p* = 0.003); 40% of confirmed cases were aged 60 years or older and a third were 41-59 years of age. Country of travel history was associated with confirmed COVID-19 (*p* ≤ 0.001) and increased likelihood of COVID-19 among the Filipino population than other nationalities in this sample (*p* = 0.024). Confirmed COVID-19 was more common among suspected cases with at least one underlying disease (*p* = 0.004), in particular, diabetes (*p* = 0.033) and those who presented with difficulty breathing (*p* = 0.033). Confirmed cases had experienced symptoms for longer (median of 7 days vs 3 days, *p* < 0.001). Among the 42 COVID-19 laboratory-confirmed cases, 9 (21%) died (data not shown). Among the 9 patients who died, the median duration of hospitalization was 5 days (0, 8) and the median duration between onset of symptoms and outcome was 11.5 days (8, 18). The sample size did not allow a detailed analysis of factors associated with an outcome of discharged or died. Among the 58 non-COVID-19 cases, 91% of the alternative diagnoses were respiratory (upper respiratory tract infection [39], community-acquired pneumonia [13], chronic bronchitis [1]). Other diagnoses included cardiac (2), viral unspecified (1), gingivitis (1), and not-specified (1).

## Discussion

In this study, we report the first 100 individuals with suspected COVID-19 admitted to an infectious disease hospital in Metro Manila from Jan. 25 to Mar. 29, 2020. For comparison, there were 2084 confirmed cases reported nationally in the Philippines as of Mar. 31, 2020 [19]. Some temporal and geographic trends can be observed, with regards to suspected and confirmed infections at SLH. The first two suspected COVID-19 cases admitted to SLH on Jan. 25, 2020, were Chinese nationals on vacation from Wuhan, becoming the first confirmed COVID-19 cases in the Philippines [6]. The Philippine government implemented travel restrictions on foreign travelers from Hubei province on Jan. 31, and then extended this to include additional countries with COVID-19 cases during February [7, 8]. The DOH conducted contact tracing on all the confirmed cases and released an interim guideline for contact tracing for confirmed COVID-19 cases on February 5, 2020 [20]. The absence of further confirmed cases among foreign nationals suggests that these measures were effective. The third confirmed case was admitted on Mar. 8, more than a month after the first case. No epidemiological link was found between the third case and the first two cases. The third, fourth, and fifth cases all reported a history of international travel. The case admitted on Mar. 10 was the first case suggestive of local transmission. On Mar. 12, the Philippine government expanded the travel ban to visitors from all 65 estimated countries with local transmission [9]. Trends toward confirmed COVID-19 cases not reporting international travel (Fig. 1) and being admitted from the populous area of Manila city rather than other areas (Fig. 2) were observed during Mar. 8-29. While only from one hospital, this data suggests the COVID-19 epidemic may have reached Manila city during this time period, as it is likely that symptomatic individuals would have attended SLH rather than another hospital. Increased COVID-19 infections in Manila city are of concern; given it is the most densely populated city in Metro Manila, with 71,263 persons per square kilometer [21]. Small dwelling sizes, social mixing due to extended families, overcrowding in slums, poses a high risk of community transmission and large outbreak in the absence of public health interventions. In order to suppress and mitigate transmission in the Philippines, the government has implemented contract tracing and surveillance, triage systems, increased testing, and improved case management. Enhanced community quarantine has been in place in Luzon island since Mar. 17, 2020 [19]. The effect of these interventions on community transmission and number of severe COVID-19 cases in the context of relatively young population in a tropical climate needs to be carefully assessed.

The factors we report to be associated with COVID-19 confirmed cases among suspected cases should be interpreted with caution. Our finding that COVID-19 confirmed cases tended to be older, Filipino, and less likely have had recent international travel history could be explained by an over-representative number of younger cases with milder symptoms in our sample, reflecting the evolving suspected case definition and referral guidelines.

In over 90% of non-COVID-19 cases, the alternative diagnosis was respiratory, highlighting the difficultly in diagnosing COVID-19 on clinical grounds and the importance of diagnostic capacity in all settings. Difficulty of breathing was the only reported symptom associated with COVID-19 infection. The presence of this symptom should alert healthcare workers to the possibility of COVID-19 infection in this setting. COVID-19 cases were more likely to have an underlying disease, in particular diabetes and hypertension. However, for the aforementioned reasons, these findings should be interpreted with caution.

Among the first 100 cases, seven health care workers were admitted, three of whom were COVID-19 confirmed. This highlights the importance of protecting HCWs during the COVID-19 pandemic. This highlights the importance of protecting HCWs during the COVID-19 pandemic. As of Apr. 6, it has been reported that 299 HCWs have tested positive and 10 have died in the Philippines [22]. During the same time period, there were reports of HCWs hit hard by COVID-19 across several countries [23]. Ensuring adequate supplies of personal protective equipment (PPE), COVID-19 testing and psychological support for HCWs will be important in order to sustain the COVID-19 response in the country. Recently, the Philippine government had secured a US$ 100 million loan from World Bank for COVID-19 Emergency Response including the provision of PPEs [24]. In addition, PPE is being locally manufactured with support from the Philippine Department for Trade and Industry [25].

The clinical characteristics of the confirmed COVID-19 cases in this study are comparable with the early case series from China and reports from other countries [4, 5, 24]. Cough and fever were the most common symptoms, similar to results reported in China, Italy, and the USA. Shortness of breath and muscle aches were less frequently reported in our study but may not have been systematically recorded on the CIF [3, 26, 27].

Among the 9 patients who died, most deaths occurred within a week of admission and within 18 days of symptom onset (median 11.5 days). A range of about 2-8 weeks from time of symptom onset to death was reported in China early in the outbreak [28]. Recent studies also report a similar range of hospitalization among the non-survivors, with the majority requiring ICU care [29, 30]. Larger adequately powered studies to analyze risk factors for mortality in this setting are required. Among admitted COVID-19 cases, the case fatality rate was 21%. This is comparable to other in-patient settings in Wuhan China (CFR—15% and 28%) and New York (CFR—21%) [26, 31, 32]. The CFR is higher than the overall mortality rate in the Philippines, reported to be 6.6% [33]. However, this cannot be compared as hospitalized cases are likely to be more severe than cases in the general population. In this study, almost half of confirmed admitted cases were older, had at least one co-morbidity, most commonly hypertension (33%), diabetes (17%), or respiratory illness (17%). Older age and hypertension were reported to be key risk factors for COVID-19 mortality in Italy [34].

This study has some limitations. First, this is a modest case series of 100 patients with suspected COVID-19 infection, of whom only 42 patients had confirmed COVID-19 infection. The study was underpowered to detect associations with mortality. Second, the findings from this study cannot be generalized to other populations in the Philippines or elsewhere given the small sample size and evolving suspected COVID-19 criteria and heterogeneous study population. Third, this analysis was limited to data collected on the CIFs and did not include detailed travel, referral or contact history, or information on investigations and treatments received. Our assumption that cases reporting recent international travel were imported may not be true and could be coincidental in some cases.

While this study provides some insights, a larger study would help further define the epidemiology and clinical features of COVID-19 in this setting. We would caution against any change in patient management based on this study. However, the data we present allows an early assessment of epidemiological and clinical characteristics of COVID-19 in Metro Manila, Philippines, and a hospital in a tropical LMIC country.

## Conclusion

As of May 2, there have been 3,267,184 cases confirmed cases globally, and 8772 confirmed cases in the Philippines [10, 35] indicating sustained community transmission. We report an increasing trend of confirmed COVID-19 cases being admitted to SLH from the densely populated Manila city area and a hospital case fatality rate of 21%.

## Data Availability

The dataset for this study is available from the corresponding author and San Lazaro Hospital on a reasonable request. Data without names and identifiers will be made available after approval from the corresponding author and San Lazaro Hospital.

## Abbreviations

CFR: Case fatality rate
CIF: Case investigation forms
COVID-19: Coronavirus disease 2019
DOH: Department of Health
HCW: Healthcare worker
LMIC: Low and middle income
NPS/OPS: Nasopharyngeal and oropharyngeal swabs
PCR: Polymerase chain reaction
PPE: Personal protective equipment
PUI: Person under investigation
RESU: Regional Epidemiologic Surveillance Unit
RITM: Research Institute for Tropical Medicine
SARI: Severe acute respiratory illness
SARS-CoV-2: Severe acute respiratory syndrome coronavirus 2
SD: Standard deviation
SLH: San Lazaro Hospital
