# Correction to: Epidemiological and clinical characteristics of patients with suspected COVID-19 admitted in Metro Manila, Philippines

**DOI:** 10.1186/s41182-020-00244-5

**Published:** 2020-07-07

**Authors:** Eumelia P. Salva, Jose Benito Villarama, Edmundo B. Lopez, Ana Ria Sayo, Annavi Marie G. Villanueva, Tansy Edwards, Su Myat Han, Shuichi Suzuki, Xerxes Seposo, Koya Ariyoshi, Chris Smith

**Affiliations:** 1 San Lazaro Hospital, Manila, Philippines; 2 School of Tropical Medicine and Global Health, Nagasaki University, Nagasaki, Japan; 3 MRC Tropical Epidemiology Group, London School of Hygiene and Tropical Medicine, London, UK; 4 Institute of Tropical Medicine, Nagasaki University, Nagasaki, 852-8523 Japan; 5 Faculty of Infectious and Tropical Diseases, London School of Hygiene and Tropical Medicine, London, UK

**Correction to: Trop Med Health 48, 51 (2020)**

**https://doi.org/10.1186/s41182-020-00241-8**

Following the publication of the original article [1], it was noted that the light red color is missing from the histogram in Fig. 1. The corrected figure has been shown below:

**Fig. 1:**
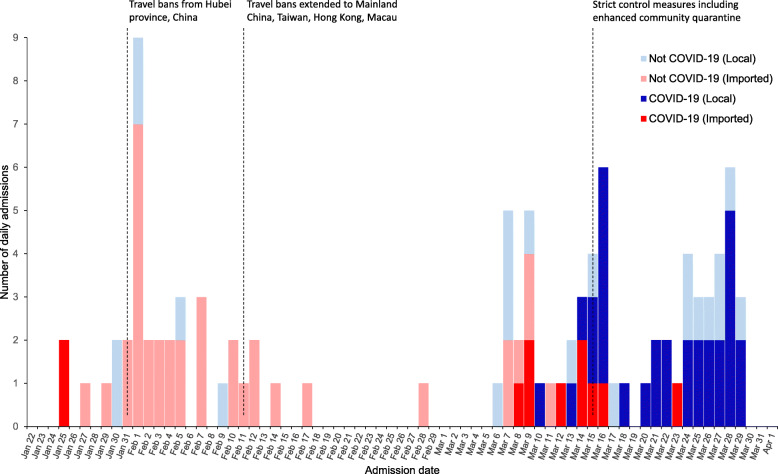
Timeline of admission date of the first 100 suspected COVID-19 cases to an infectious diseases hospital in Metro Manila. Cases were considered “imported” if a history of international travel was reported within 14 days prior to the admission, and conversely ‘local’ if no international travel was reported within 14 days prior to the admission
